# Household

**Published:** 1889-04

**Authors:** 


### HOUSEHOLD.

Lemon Pudding.—Three cups of bread crumbs, one cupful (scant) of sugar, two liberal tablespoons of corn starch, one lemon, juice and rind, two cups of milk, one heaping teaspoon of butter ; scald the milk and stir in the corn starch wet up in four teaspoons of cold water. Cook, stirring all the time, until it thickens well; add the butter and set aside until cold ; beat the eggs light, and the sugar, the lemon juice and grated peel, and whip in, a large spoonful at a time, the stiffened corn starch milk. Bake in a buttered dish.

Fried Chicken.—Cut the chicken in pieces, lay it in salt water, which change several times; roll each piece in flour; fry in very hot lard or butter; season with salt and pepper; fry parsley with them also. Make a gravy of cream seasoned with salt, pepper and a little mace, thicken with a little flour in the pan in which the chickens were fried, pouring off the lard. Use a granite iron fry pan.

Sauce for Boiled Fish.—To one teacup of milk add one teacup of water; put it on the fire to scald, and when hot stir in a tablespoon of flour, previously wet with cold water; add two or three eggs; season with salt and pepper, a little celery, vinegar and three tablespoons of butter. Boil four or five eggs hard, take off the shells and cut in slices and lay over the dish. Then pour over the sauce and serve.

The Coffee Pot.—It seems a very simple thing to make good coffee, and yet in no one thing do so many housekeepers fail; the coffee pot, however, is largely responsible for the failure. Many housekeepers who have servants think the cook will not fail to attend to the cleaning of the coffee pot, while others who do their own work attach no importance to the matter, and allow the grounds and cold coffee to stand in it for several days without emptying, and at the same time wonder why they never have good coffee, notwithstanding the best article is bought, well parched and carefully made. To have the beverage perfect it is necessary that great attention be given the coffee pot. It should be emptied after each meal, well washed inside and outside in clean, hot soap-suds, then rinsed in boiling water first and then in cold water, after which it should be dried and set in the sun. Occasionally, (once a week) it is well to put a tablespoonful of soda in the coffee pot, fill with boiling water and set on the stove. This cleanses and purifies it. This attention to what may seem a very small matter, is of the utmost importance to the housekeeper, and will be found well worth the slight extra trouble by all who try it. After a good article of coffee, nothing, then, is so important, in order to have good coffee, as a clean coffee pot.

Eggs.—The Spaniards have three hundred and sixty-five ways of cooking eggs, but anyone that can boil, fry, scramble and poach an egg to perfection, and top off by learning to make a good omelet, can let all the rest of the ways go. Eggs, like milk, are a perfect food. A two-ounce egg contains nearly the same amount of nourishment as an ounce of meat and an ounce of bread. Even if sold at sixty cents a dozen, says Marion Harland, they are the cheapest kind of food for breakfast and lunch; they ought to be on our tables every day.

